# Investigating the Clinical Utility of the Anti-Mullerian Hormone Testing for the Prediction of Age at Menopause and Assessment of Functional Ovarian Reserve: A Practical Approach and Recent Updates

**DOI:** 10.14336/AD.2021.0825

**Published:** 2022-04-01

**Authors:** Fahimeh Ramezani Tehrani, Faezeh Firouzi, Samira Behboudi-Gandevani

**Affiliations:** ^1^Reproductive Endocrinology Research Center, Research Institute for Endocrine Sciences, Shahid Beheshti University of Medical Sciences, Tehran, Iran.; ^2^Pathology Department, Shahid Beheshti University of Medical Sciences, Tehran, Iran.; ^3^Faculty of Nursing and Health Sciences, Nord University, Bodø, Norway.

**Keywords:** anti-müllerian hormone, functional ovarian reserve, time to menopause

## Abstract

Low ovarian reserve is a serious condition, leading to sterility in up to 10% of women in their mid-thirties. According to current knowledge, serum anti-Müllerian hormone (AMH) levels for age are the best available marker for the screening the quantity of a woman’s functional ovarian reserve, better than age alone or other reproductive markers. This review summarizes recent findings, clinical utility and limitations in the application of serum AMH testing as an accurate marker for the screening of functional ovarian reserves and predicting age at menopause. AMH assessment hold promise in helping women make informed decisions about their future fertility and desired family size. However, screening of the functional ovarian reserve could be offered to all women at 26 years of age or older who seek to assess future fertility or in case of personal request, ovarian reserve screening may be considered beyond 30 years; however, it has never been advocated beyond 35 years, since it is not advisable to delay childbearing beyond this age. In this respect, an age-specific serum AMH levels lower than the 10th percentile may be used as a threshold for the identification of a low functional ovarian reserve in an individual woman. Its level should be interpreted with caution in the adolescent and young women aged below 25 years (since AMH levels peak at this age); recent users of hormonal contraceptives (since AMH levels transiently decrease until two months after discontinuation); and women with PCOS (which dramatically increases AMH levels). However, the ability of AMH levels to predict the time to menopause is promising but requires further investigation and routine AMH testing for the purposes of predicting the time to menopause is not recommended.

Societal shifts triggered by a greater focus on education and career opportunities, developments in assisted reproduction, rise of effective contraception, value changes, gender equity, partnership changes, lack of suitable partners, housing conditions, economic uncertainty, and the absence of supportive family policies have resulted in a trend toward delayed childbearing worldwide [1]. It has been estimated that over 5% of pregnancies in Western countries have been occurred after the fourth decade of life, which the rate has been more than doubled in recent decades [2, 3].

Assessment of functional ovarian reserves hold promise in helping women make informed decisions about their future fertility and desired family size, rather than simply providing them with generic age-related fertility recommendations [4-6].

Here, we discuss the clinical utility and limitations of anti-Müllerian hormone (AMH) testing as an accurate marker for the screening of functional ovarian reserves and predicting age at menopause.

## The functional ovarian reserve

The functional ovarian reserve defines the quantity and quality of the ovarian primordial follicular pool, which declines with age in women, with a more rapid decline after the mid-30s until it reaches undetectable levels at menopause [7]. Although age is known to be the most important factor affecting the quality of the ovarian reserve, [8, 9] other intrinsic and extrinsic factors also play a role (Table 1) [10-21]. Therefore, women of the same age can vary in their fertility potential.

**Table 1:** Factor associated with ovarian reserve.

Factors	Level of evidence
Intrinsic factors	
Genetic	
Individual variation	2a
BRCA carrier,	2b
FMR1 premutation	2c
Fragile X	3a
Turner syndrome	3a
Race and Ethnicity	2c
Age	2a
Family history of POI or early menopause	2a
Extrinsic factors	
Unhealthy lifestyle factors	
Heavy and current smoking	3b
Frequent binge drinking	3b
Vitamin D deficiency	2b
Body mass index (obesity)	3a
Medications	
Immunosuppressive	3b
Chemotherapy drugs	2b
Ovarian Pelvic surgery	2b
Acute or chronic psychosocial stress	3b
Some Systemic or Autoimmune Disorders	3b

BRCA-1, breast cancer gene-1; FMR1, POI, Premature Ovarian insufficiency; 2a, Systematic reviews (with homogeneity) of cohort studies; 2b, Individual cohort study or low quality randomized controlled trials; 2c, "Outcomes" Research; ecological studies; 3a, Systematic review (with homogeneity) of case-control studies; 3b, Individual case-control study.

Low ovarian reserve is a serious condition, leading to sterility in up to 10% of women in their mid-thirties, and is mostly irreversible [22]. The condition is often clinically asymptomatic, making it difficult to diagnose until fertility is diminished, and therefore, ovarian reserve screening could be offered to all women ≥26 years old who seek to assess future fertility [22]. However, since the woman's fertility start declining in their thirties, it is not recommended to postpone fertility after those ages, particularly for women who have never conceived before which have a lower probability of achieving a pregnancy in later life. However, in case of personal request, ovarian reserve screening may be considered beyond 30 years; however, it has never been advocated beyond 35 years, since it is not advisable to delay childbearing beyond this age [22].

Early diagnosis is critical to help women make informed decisions about their plans for natural conception, opt for oocyte vitrification, or use donated sperm if single, while these alternatives still have reasonable prospects of success [8]. Although no marker exists that directly measures the ovarian reserve, the size of the growing follicle pool remaining in the ovary is precisely detectable by some biomarkers, and this measure is referred to as the ‘functional ovarian reserve’ [8, 23, 24].

Over the years, numerous parameters have been proposed to estimate the functional ovarian reserve. Serum follicle-stimulating hormone (FSH) and antral follicle count (AFC) have been widely applied in reproductive medicine for years. Beyond their general predictive value, both have specific disadvantages. FSH levels fluctuate during the inter/intra cycle of menstruation; tend to increase after 35 years of age; are dependent on the functional hypothalamic-pituitary-ovarian (HPO) axis; and have low sensitivity when the number of follicles significantly decreases. AFC has various sensitivities owing to technical limitations such as interobserver variability and ultrasound machines, which are restricted to antral follicles of measurable size; has different methodologies for counting antral follicles and intercycle variation in obese women; and may not be feasible for virgin girls in some cultures, if using the vaginal approach. Compared to these other parameters, serum AMH is currently the best proxy for assessing the ‘functional ovarian reserve’, which supports normal follicle progression and ovulation [9-13].

## What is Anti-Müllerian hormone?

**Figure 1.**
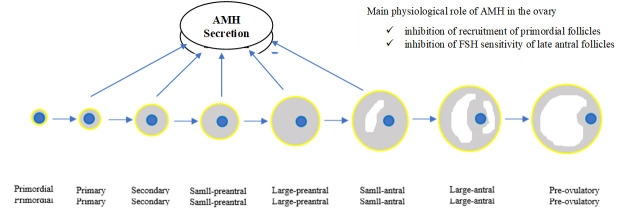
Schematic model of AMH production and its role in the ovary.

Anti-Müllerian hormone is specifically expressed by small growing follicles in the ovary and has an inhibitory effect on primordial follicular recruitment and FSH-induced preantral follicle growth (Fig. 1) [25]. From birth onwards, AMH levels increase steadily till 9 years of age, decline slightly during the pubertal ages, and peak at around 25 years of age. This is followed by a gradual decline brought about by the reduction of the primordial follicle pool with age and until it reaches undetectable levels at an average of 50-51 years of age, corresponding to menopause [26]. In addition, AMH has several other unique characteristics that, taken together, make it a reliable marker for the assessment of the functional ovarian reserve and time to menopause, better than age alone or other reproductive hormones. The important characteristics are that it is secreted exclusively by ovarian follicles at approximately constant levels from one cycle to another, with a consistent pattern throughout each menstrual cycle, particularly after the age of 30 years. Therefore, measurements can be taken at any stage of the menstrual cycle. It also produces a reliable estimate with a single measurement and is largely independent of hypothalamic pituitary function. Further, the test is the least expensive and intrusive of the options available and also has the least inter-observer variability [26, 27].

Its utility is further extended as a marker of ovarian responsiveness in assisted reproduction, ovulatory disorders such as polycystic ovary syndrome (PCOS), before or after ovarian damage by surgery, chemotherapy, or radiotherapy, as well as prediction and diagnosis of early menopause and premature ovarian insufficiency. In addition, given the specific site of production of AMH, its measurement has been used as a very precise marker of tumours originating from granulosa cells in female and Sertoli cells of the testis in males [28]. Moreover, it may be a good candidate marker for obtaining information on testicular function and spermatogenesis in disorders related to male fertility [29].

## What is the role of AMH in the assessment of low functional ovarian reserves?

According to current knowledge, age-specific serum AMH levels, which are measured by Automated and therefore more accurate AMH assays [30], are the best available marker for the assessment of the functional ovarian reserves, better than age alone or other reproductive markers [8]. However, for clinical interpretation, the prediction of age at menopause (as the most important indicator for ovarian aging), that been presented according to various ages and AMH percentiles, can be used [31-32]. For instance, an age-specific AMH level lower than the 10th percentile may be used as a threshold for the identification of those with low functional ovarian reserve [22]. For example, a 30-yr-old woman with an AMH value less than 0.5 ng/ml has diminished functional reserve and is at risk of early menopause [31,32]. These specific values should be confirmed with further comprehensive studies.

• In cases of diminished functional ovarian reserves, individual risk assessment should be performed based on repeat confirmatory AMH testing, day 3-5 FSH measurement, and ultrasound findings of AFC. Final diagnosis should be made after complete evaluation of all results, in the context of any known individual risk factors for low ovarian reserves [22]. Women with low ovarian reserves who are interested in childbearing should be encouraged to become pregnant spontaneously within the next 6 months. If natural conception does not occur within that time, assisted reproduction should be considered. However, since fertility is associate with numerous factors, the ability of AMH to predict the chance of pregnancy is less promising, and AMH should not be used as a fertility test.
• In women with borderline diminished functional ovarian reserves, annual follow-up ovarian reserve testing is recommended. In addition, the restriction of factors that can increase the rate of decline, including impacts of certain lifestyles such as alcohol abuse, smoking, malnutrition, obesity, and stress should be highlighted.
• In women with acceptable AMH levels, it should be emphasised that advanced maternal age is related to low quality of oocytes and is therefore strongly associated with an increased risk of aneuploidy and other adverse feto-maternal or neonatal outcomes.

It is important to remember that individual conditions can affect AMH values, and these should be considered prior to their clinical application and interpretation. Serum AMH levels do not peak until approximately 25 years of age; hence, care must be taken when interpreting AMH levels in adolescents and young women. In addition, hormonal contraceptive usage, particularly in long-term users, could transiently decrease serum concentrations of AMH by 20-30% but tend to recover after two months of discontinuation. Therefore, AMH should be measured at least 2 months after the discontinuation of hormonal contraceptives. Moreover, due to an increase in the number of pre-antral and small antral follicles, which primarily produce AMH, the serum concentration of AMH tends to be higher in women with PCOS compared to non-PCOS counterparts. Although this needs to be precisely defined, [33] the threshold levels for the prediction of PCOS in the age categories of 20-27, 27-35, and 35-40 years could be 5.7 (95 % CI: 5.48-6.19), 4.55 (95 % CI: 4.52-4.64), and 3.72 (95 % CI: 3.55-3.80), respectively, which all have positive predictive values (PPV) of more than 90% and negative predictive values of more than 80% [23-26, 34].

Finally, women must be empowered to make voluntary and informed choices about whether to get tested based on objective information about all aspects of this test. Healthcare professionals may tend to focus on the implications of poor results, but the information provided should also include full aspects of both poor or acceptable test results and subsequent steps of action prior to the test is ordered.

## What is the role of AMH in predicting the age at menopause?

Age-specific AMH levels may provide a fairly reasonable prediction of age at menopause (natural or iatrogenic), particularly for women who are younger than 40 years, even if they still have regular menstrual cycles and normal levels of other reproductive hormones [9, 22]. It could therefore provide increasing clinical value to young women who are at risk of early menopause due to genetic, familial, and environmental factors or medical intervention [25, 33] in cancer survivors both before and after gonadotoxic treatment [15].

Mathematical modelling has already been used to predict age at menopause. For example, a 30-year-old woman with an AMH level close to 1.1 ng/mL (7.85 pmol/L) is known to have a predicted mean age at menopause of 45 (range, 37-50) years [31]; a serum level of AMH less than 0.1 ng/mL (0.71 pmol/L) in any reproductive age was strongly associated with the imminent onset of menopause [31-39]. Women at risk should be managed by proper counselling about future fertility and other early or late menopause-related problems such as cardiovascular disease, osteoporosis, and breast and endometrial cancers [40].

However, based on available studies (Table 2) as well as available meta-analysis [41], the precise predictive value of serum age-specific AMH levels for time to menopause, remains controversial, since the majority of available studies have assessed regularly cycling women of various ethnicities, age ranges, duration of follow-up, and AMH assays. Moreover, the trajectory of the decline also seems to differ among women [42]. More controversies are observed when ‘extreme ages at menopause’, including early menopause [41] and premature ovarian insufficiency (POI), are predicted by AMH levels, and for such women, the accuracy of this prediction is more critical. Poor accuracy is mainly due to low sample sizes of women extreme ages at menopause in available cohorts, and by adding ongoing cohorts, the reliability of statistical models for prediction of POI should be improved [43]. At the current time, the clinical application of AMH levels for the prediction and diagnosis of menopause, particularly early menopause or POI, is promising but needs further investigations.

**Table 2:** Summary of some important studies used AMH for prediction of menopause.

Author	Country	Type of study	Participants characteristics at initiation of study	Number of women reached menopause throughout the study	Average follow-up time	Assay kit	Findings
Bertone-Johnson et al. 2018 (46)	USA	Nested case-control within the prospective study (Nurses’ Health Study II)	Cases: N = 327, Age: 40.2 (2.8), BMI: 25.3 (0.3) Matched Controls: n = 327, Age: 40.2 (2.8), BMI: 25.0 (0.3)	-	12 years	Pico AMH	Each 0.10 ng/mL (0.71 pmol/L) decrease in AMH was associated with a 14% higher risk of early menopause.
Broer et al. 2011 (47)	Netherlands	Follow-up study at an academic hospital	N = 257, Age: 35.5 (5.9), BMI: 24.0 (4.0)	48 (18.7%)	11 years	DSL and Gen-II ELISA	AMH is capable of predicting future age at menopause for a given woman
de Kat et al. 2016 (42)	Netherlands	Population-based cohort study (Doetinchem study)	N = 3133, Age: 40 (10), BMI: 25 (4.0)	1882 (60.1%)	20 years	picoAMH	There is no fixed pattern for decline rate of AMH and the difference between women with high and low age-specific AMH levels decrease as age progresses.
de Kat et al. 2019 (48)	Netherlands	Population-based cohort study (Doetinchem study)	N = 2434, Age: 36.1 (8.1), BMI: -	1298 (53%)	11.6 years	picoAMH	Knowledge of the AMH decline rate does not improve the prediction of menopause and early menopause.
Depmann et al. 2016 (49)	Netherlands	Population-based cohort study (Doetinchem study)	N = 216, Age: -, BMI: -	81 (37.5%)	14.8 years	DSL and Gen-II ELISA	AMH alone predicts age at menopause; however, its predictive value decreased with increasing age of the woman
Dólleman et al. 2015 (50)	Netherlands	Population-based cohort study (Doetinchem study)	N = 1163, Age: 40.8 (7), BMI: 23.8 (3.9)	-	10 years	Gen-II ELISA	AMH has additive predictive value for prediction of age at menopause even when taking age, BMI, cycle regularity and smoking into account.
Finkelstein et al. 2020 (51)	USA	Prospective cohort study (Women's Health Across the Nation)	N = 1537, Age: 47.5 (2.6), BMI: 23.8 (3.9)	-	Until 12 months of amenorrhea occurred all participants	picoAMH	Using an ultrasensitive ELISA with a limit of detection of 1.85 pg/ml, is clinically useful for predictions of the time to menopause.
Freeman et al. 2012 (52)	USA	Population-based cohort study (Penn Ovarian Aging Study)	N = 293, Age *: 40.93 (40.6 -41.3), BMI: -	146 (50%)	14-year	Gen-II ELISA	The AMH decline rate of change increases the precisions of the estimation of time to menopause, when included with an AMH baseline level and age, in late reproductive-age women.
Freeman et al. 2012 (53)	USA	Population-based cohort study (Penn Ovarian Aging Study)	N = 401, Age *: 41.47 (41.13- 41.82), BMI *: 29.33 (28.56 -30.10)	198 (49.4%)	14-year	Gen-II ELISA	Among women with a baseline AMH level below 0.20 ng/mL (1.42 pmol/L), the median time to menopause was 5.99 y, in the 45- to 48-yr age group and 9.94 y in the 35- to 39-y age group.
Gohari et al. 2016 (54)	Iran	Population-based cohort study (Tehran Lipid and Glucose Study)	N = 266, Age: 37.55 (9.61), BMI: 27.7 (4.99)	63 (23.7%)	6.5 years	DSL	Decline rate of AMH is specific for each woman and could predict age at menopause.
Kim et al. 2017 (9)	USA	Population based cohort study (Coronary Artery Risk Development in Young Adults study)	N = 426, Age: 43 (39-45), BMI: 28 (24-34)	55 (13%)	5 years	Pico AMH	The majority of women aged 45-49 with AMH values <0.02 ng/dl underwent menopause within 5 years.
La Marca et al. 2013 (55)	Italy	Cross-sectional study	N = 375, Age: 35.3 (0.2), BMI: 23.2 (4.2)	-	-	Gen-II ELISA	There were the good level of conformity between the distributions of observed and AMH-predicted ages at menopause. Using BMI and smoking status as additional variables improves AMH based prediction of age at menopause.
Nair et al. 2015 (56)	USA	Population based cohort study (Coronary Artery Risk Development in Young Adults study)	N = 716, Age: 42, BMI: 27.8	207 (29%)	9 years	Pico AMH	AMH appears identified women at risk of menopause in the near future, within 3 years of AMH measurement. The risk of menopause was over 6-fold higher for a 0.5 ng/dL (3.57 pmol/L) decrement in AMH.
Ramezani Tehrani et al. 2009 (57)	Iran	Population-based cohort study (Tehran Lipid and Glucose Study)	N = 147, Age: 44.8 (2.6), BMI: 28.9 (4.6)	60 (40.8%)	6 years	DSL	Single AMH measurement is a good predictor for the onset of menopause. Of every 10 women who are naturally fertile, aged 40 to 50 years with a normal menstrual cycle at the time of the test, will not reach menopause status within the next 6 years if the AMH level is greater than 0.39 ng/mL (2.78 pmol/L).
Ramezani Tehran et al. 2020 (58)	Iran	Population-based cohort study (Tehran Lipid and Glucose Study)	N = 959, Age: 36 (7.1), BMI: 27 (4.7)	529 (55.2%)	14 years	Gen-II ELISA	Prediction of age at menopause could be improved by multiple AMH measurements. On average for the same amount of age-specific AMH, the predicted age at menopause for those with the highest AMH decline rate (95^th^ percentiles) was about one decade lower than those with the lowest (5th percentiles).
Ramezani Tehran et al. 2011 (59)	Iran	Population-based cohort study (Tehran Lipid and Glucose Study)	N = 266, Age: 37.6 (9.6), BMI: 27.7 (5.0)	63 (23.7%)	6 years	DSL	Ages at menopause for different levels of serum AMH concentration among women aged 20 to 49 years has been presented.
Ramezani Tehran et al. 2013 (60)	Iran	Population-based cohort study (Tehran Lipid and Glucose Study)	N = 1015, Age: 36.7 (7.5), BMI: 27.1 (4.7)	277 (27.3%)	10 years	Gen-II ELISA	Average age at menopause for individual women aged 20 to 49 years for various amount of AMH is presented.
van Disseldorp et al. 2008 (61)	Netherlands	Nested cross-sectional study (European Prospective Investigation into Cancer and Nutrition study)	N = 144, Age: 37.9 (5.5), BMI: 24.2 (3.8)	-	-	DSL	There was good conformity between the observed distribution of age at menopause and that predicted from declining AMH levels
van Rooij et al. 2004 (38)	Netherlands	Prospective cohort study	N = 81, Age: -, BMI: -	-	4 years	Pico AMH	AMH, had the high predictive accuracy for occurrence of cycle irregularity and could predictor for the occurrence of menopausal transition within 3 to 5 years

## What techniques can be used to measure the AMH?

There were different commercial enzyme-linked immunosorbent assay (ELISA) kits used to measure AMH, which is varied in antibody pairs, standard curve ranges, and limits of detection [44] (Table 3). Assays for AMH have progressed over the years, which is the major step forward for clinical epidemiological use. However, despite the current progress, universally accepted and standardized materials and method of assay to measure the serum concentrations of AMH are still lacking. Meanwhile, there are not conversion protocol to determine comparability of AMH assays available to facilitate the interpretation of values obtained in different studies. Both needs to be defined precisely for any AMH clinical use. However, a picoAMH assay with a lower detection threshold from Ansh Laboratories are most sensitive assay for detection of low levels of AMH [45].

## What are the limitations of AMH testing?

AMH has the following limitations:

• The age-dependent decline rate of AMH levels varies among women. An international guideline regarding age-specific AMH diagnostic thresholds for screening functional ovarian reserves or predicting age at menopause remains to be addressed.
• Some endogenous and exogenous factors could influence serum AMH levels, which limit the accurate interpretation of AMH values in a clinical setting.
• The predictive value of AMH for successful clinical pregnancy (both in natural and assisted reproduction) is less promising; and therefore, AMH levels should not be used as a fertility test.
• AMH assessment using manual enzyme-linked immunosorbent assay (ELISA) has some limitations due to intra-assay/interassay differences and requires careful sample preparation and storage. However, automated AMH assay platforms have greater precision, faster turnaround time, greater sensitivity, and accessibility in many countries and should be used as a standard method for AMH measurement.

**Table 3:** Commercial assays available for AMH measurement.

AMH assay, year	Manufacture company	Detection limit	Standard curve range	description
IOT, 1999	Immunotech	0.05 ng/mL	0.1-24.5 ng/mL	A monoclonal antibody pair were directed, one directed at the pro region and the other at the mature region.
DSL, 2003	Diagnostic Systems Lab-oratories	0.006 ng/mL	0.05-15 ng/mL	Both monoclonal antibodies were directed at the mature region
Gen II generation, 2010	Beckman Coulter	0.16-22.5 ng/mL	0.08 ng/mL	The DSL antibodies were used in assay, which was standardized to the IOT assay
Ultrasensitive, 2012	Ansh Labs	0.083-14.2 ng/mL	0.023 ng/mL	Monoclonal antibody pair directed against specific linear epitopes in the stable pro region and mature region of the associated form of human recombinant AMH
PicoAMH, 2013	Ansh Labs	0.001-0.746 ng/mL	0.001 ng/mL	

In conclusion, considering the precise results, the ease of a serum-based test, independent of specific time in the cycle serum, independent of hypothalamic pituitary function and very low inter-observer variability, AMH is the preferred ovarian reserve marker. However, lack of international guideline regarding age-specific AMH diagnostic thresholds for screening functional ovarian reserves or predicting age at menopause could limit the proper interpretation of AMH values in a clinical setting and remain to be addressed in future researches.

Ultimately, further research is needed to understand whether releasing/restricting various factors that could affect ovarian status and also the optimal approaches that promote ovarian reserve among women.
